# Atrial fibrillation hemodynamic effects on lenticulostriate arteries identified at 7‐Tesla cerebral magnetic resonance imaging

**DOI:** 10.1002/ctm2.1367

**Published:** 2023-09-21

**Authors:** Andrea Saglietto, Stefania Scarsoglio, Francesco Tripoli, Jaco Zwanenburg, Geert Jan Biessels, Gaetano Maria De Ferrari, Luca Ridolfi, Matteo Anselmino

**Affiliations:** ^1^ Division of Cardiology, Cardiovascular and Thoracic Department “Città della Salute e della Scienza” Hospital Turin Italy; ^2^ Department of Medical Sciences University of Turin Turin Italy; ^3^ Department of Mechanical and Aerospace Engineering Politecnico di Torino Turin Italy; ^4^ Center for Image Sciences University Medical Center Utrecht Utrecht The Netherlands; ^5^ Department of Neurology, UMC Brain Center University Medical Centre Utrecht Utrecht The Netherlands; ^6^ Department of Environmental, Land and Infrastructure Engineering Politecnico di Torino Turin Italy

Dear Editor,

Over the past 20 years, atrial fibrillation (AF), the most common tachyarrhythmia, has been associated with an increased risk of cognitive decline/dementia, which appears to be independent from overt clinical cerebrovascular events.^1^ In particular, AF patients often present multiple, apparently silent cerebral lesions, which relate to reduced cognitive function. These ‘silent’ lesions are frequently found at the subcortical level and belong to the cerebral small vessel disease (SVD) spectrum (lacunar infarctions, white matter lesions and microbleeds), thus a cardioembolic genesis for these lesions is unlikely. One of the main vascular supplies to subcortical and white matter areas is provided by lenticulostriate arteries (LSAs), small arteries perpendicularly departing from anterior (ACA) and middle (MCA) cerebral arteries. We hypothesize that AF, by its ‘irregularly irregular’ rhythm, may exert direct detrimental beat‐to‐beat hemodynamic effects at the LSA level. To explore this hypothesis, we performed a computational fluid dynamics (CFD) analysis simulating AF and sinus rhythm (SR) on LSA segmentations derived by 7T high‐resolution cerebral magnetic resonance imaging (MRI).

A detailed description of the Methods can be found in the Supporting Information and in a previous publication by our group,^2^ where an advanced CFD approach was applied on the vascular morphology of a single subject, suggesting that AF may increase the atheromatosis and thrombogenesis risks in the LSAs region. Briefly, a total of 17 LSA geometries (ranging from 1 to 8 geometries per each of the 10 healthy subjects enrolled — please refer to Supporting Information for main clinical characteristics) were segmented from raw MRI data, the majority originating from the MCA (right MCA: 7/17, 41%; left MCA: 7/17, 41%; ACA: 3/17, 18%). Wall shear stress (WSS) range (Δ𝑊𝑆𝑆) and intraluminal pressure range (Δ*P*) were computed on all the 17 LSA segmented geometries, both in AF and in SR conditions. Ranges correspond to the difference between the WSS and P magnitude obtained in correspondence of the 95th percentile of the beat‐to‐beat maximum flows and that at the 5th percentile of the beat‐to‐beat minimum flows.

As shown in Figure 1A (left panel), the highest Δ𝑊𝑆𝑆 was computed at the proximal portion of the LSAs (region of interest, ROI, 2), just after the origin of the vessel (ROI2 centreline length: .88 ± .1 mm), in both simulated rhythms. AF, compared to SR, induced higher Δ𝑊𝑆𝑆 in this critical anatomical site (4.26 ± 2.71 Pa vs. 3.07 ± 1.91 Pa, *p* < .001; relative difference: +38.76%). Of note, the area with the highest shear stress was .57 ± .2 mm^2^ in AF, significantly wider than in SR (.26 ± .14 mm^2^; *p* < .001). Figure 1A (right panel) highlights, in the same LSA, the AF‐SR relative Δ𝑊𝑆𝑆 difference, further underlining that the origin of the vessel was the site where AF, compared to SR, increased Δ𝑊𝑆𝑆 the most. Figure 1B graphically displays mean Δ𝑊𝑆𝑆 at ROI2, together with the relative difference between AF and SR, in each specific anatomical model. Δ𝑊𝑆𝑆 for ROI2 in all the 17 LSA models, both in SR and AF conditions, are reported in Figure S4. Concerning Δ*P*, its value along the anatomical course of the vessel (ROI1→ ROI10) was higher in AF than in SR (ROI10: 62.55 ± .95 mmHg vs. 50.37 ± .69 mmHg, *p* < .001; relative difference: +24.18%), with the origin of the vessel, similarly to Δ𝑊𝑆𝑆, being both the most stressed region (higher Δ*P*) and the site showing the maximum relative difference between AF and SR conditions (Figure 2A). Figure 2B graphically displays the mean Δ*P* across each specific anatomy, together with the relative AF−SR difference.

**FIGURE 1:**
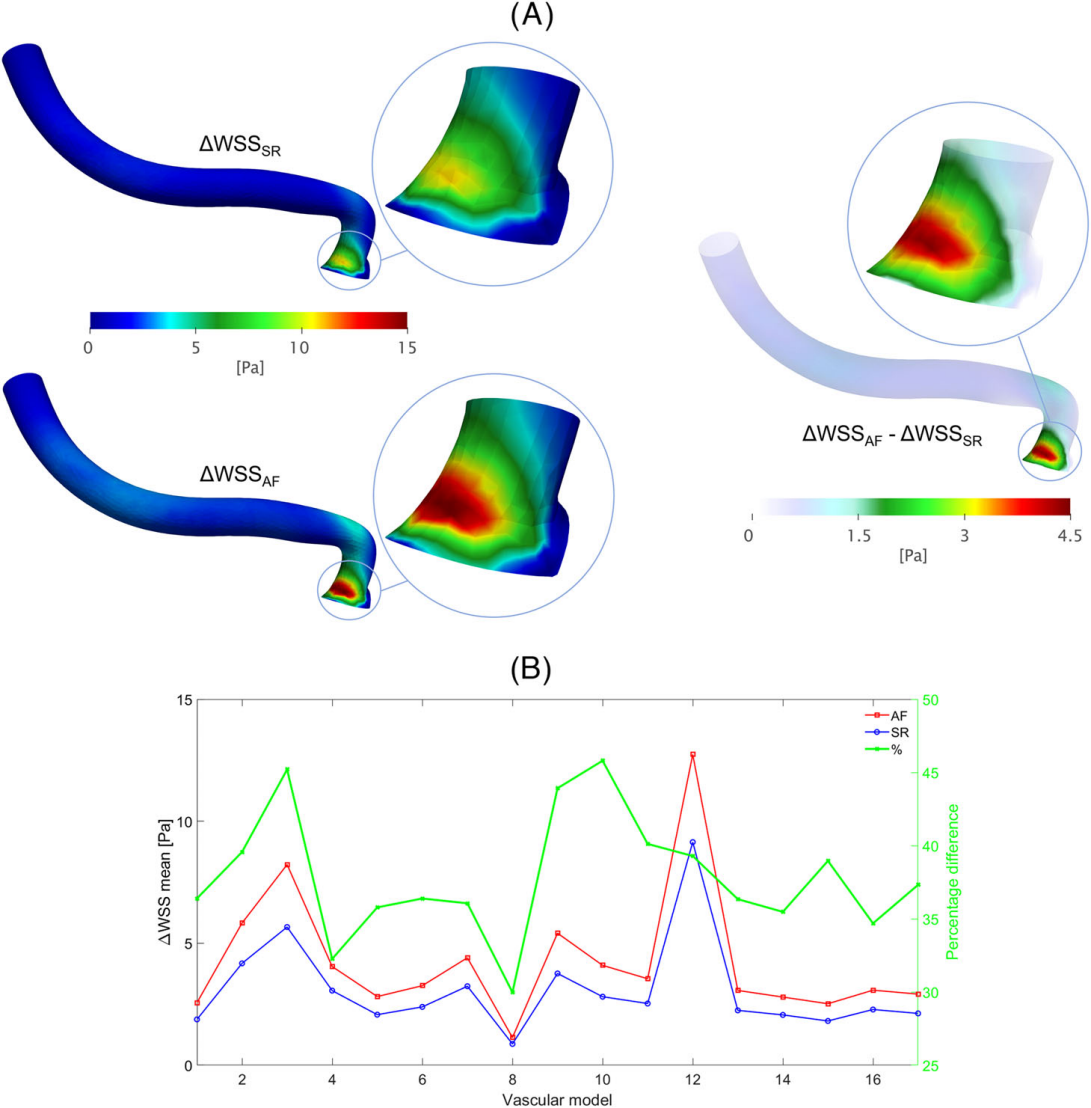
(A) CFD models of a representative LSA displaying local Δ𝑊𝑆𝑆 values (left panel: SR and AF; right panel: AF−SR relative difference) along the entire 3D segmentation and in the ROI with *n* = 2 (zoomed inlets); (B) Δ𝑊𝑆𝑆 at the proximal portion (ROI2) of each 3D anatomy in the two rhythm conditions (AF and SR) and corresponding relative difference (using SR values as baseline).

**FIGURE 2:**
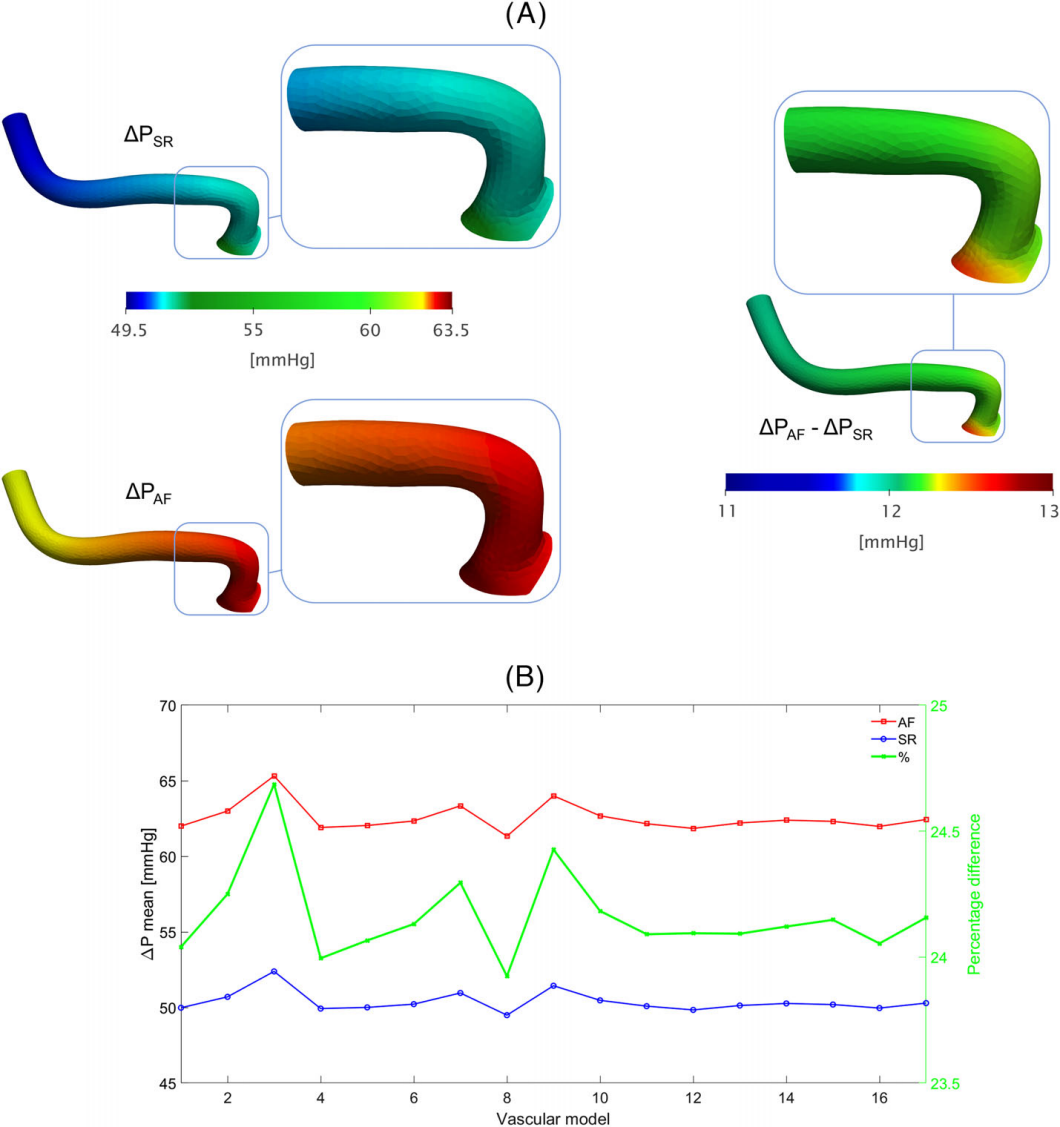
(A) CFD models of a representative LSA displaying local Δ*P* values (left panel: SR and AF; right panel: AF−SR relative difference) along the entire 3D segmentation and in the ROI with *n* = 10 (zoomed inlets); (B) Δ*P* values (at ROI10) for each 3D anatomy in the two rhythm conditions (AF and SR) and corresponding relative difference (using SR values as baseline).

Overall, the present analysis clearly indicates that AF, compared to SR, exposes LSAs to an increased range of WSS, particularly at their origin, and to wider ranges of intraluminal pressure along the vessels. In this regard, the SWISS‐AF study,^3^ in a group of properly anticoagulated AF patients without a history of clinical cerebrovascular accidents, revealed at cerebral MRI silent cerebral lesions in approximately 20% of the patients, being white matter lesions and microbleeds the most common pattern of multiple lesion type.^4^ While a subclinical cardioembolic genesis is reasonable for the cortical location, this mechanism seems unlikely for lesions belonging to the cerebral SVD spectrum, such as lacunar infarctions, white matter hyperintensities and microbleeds. An intriguing alternative hypothesis is that these lesions might be promoted by hemodynamic alterations induced by the typical ‘irregularly irregular’ AF pattern. In fact, in the last few years, scientific evidence has accumulated suggesting that AF reduces mean cerebral blood flow^5^ and that repetitive extreme hemodynamic events, occurring during the arrhythmia, result in transient critically reduced perfusion or pressure bursts in the distal cerebral circle.^6^, ^7^ Present data further reinforce this hypothesis by highlighting how AF may also directly affect local hemodynamics of LSAs and by providing a plausible framework explaining the contribution of AF, on top of other known risk factors, such as hypertension, diabetes mellitus, smoking and hypercholesterolemia,^8^ in the genesis of non‐cardioembolic silent cerebral lesions (Figure 3). While normal shear stress oscillations are known to be athero‐protective, excessively low values are pro‐atherogenic (considering that local low shear stress environments lead to atherosclerotic plaque development and progression^9^), and excessively high values induce plaque erosion and rupture.^10^ A disproportionate shear stress variation, may, therefore, relate to the higher propensity of an AF patient to activate an atherosclerotic process at the LSA origin, facilitating the proximal occlusion of the vessel. In addition, AF exposes LSAs to increased pressure ranges. In response to transient, repetitive exposure to high blood pressure levels, LSAs more easily suffer arteriolosclerosis and lipohyalinosis, eventually facilitating lacunar strokes^11^ or blood−brain barrier damage.^8^ On the other hand, repetitive exposure to transient low blood pressure facilitates downstream hypoperfusions.^6^, ^7^

**FIGURE 3:**
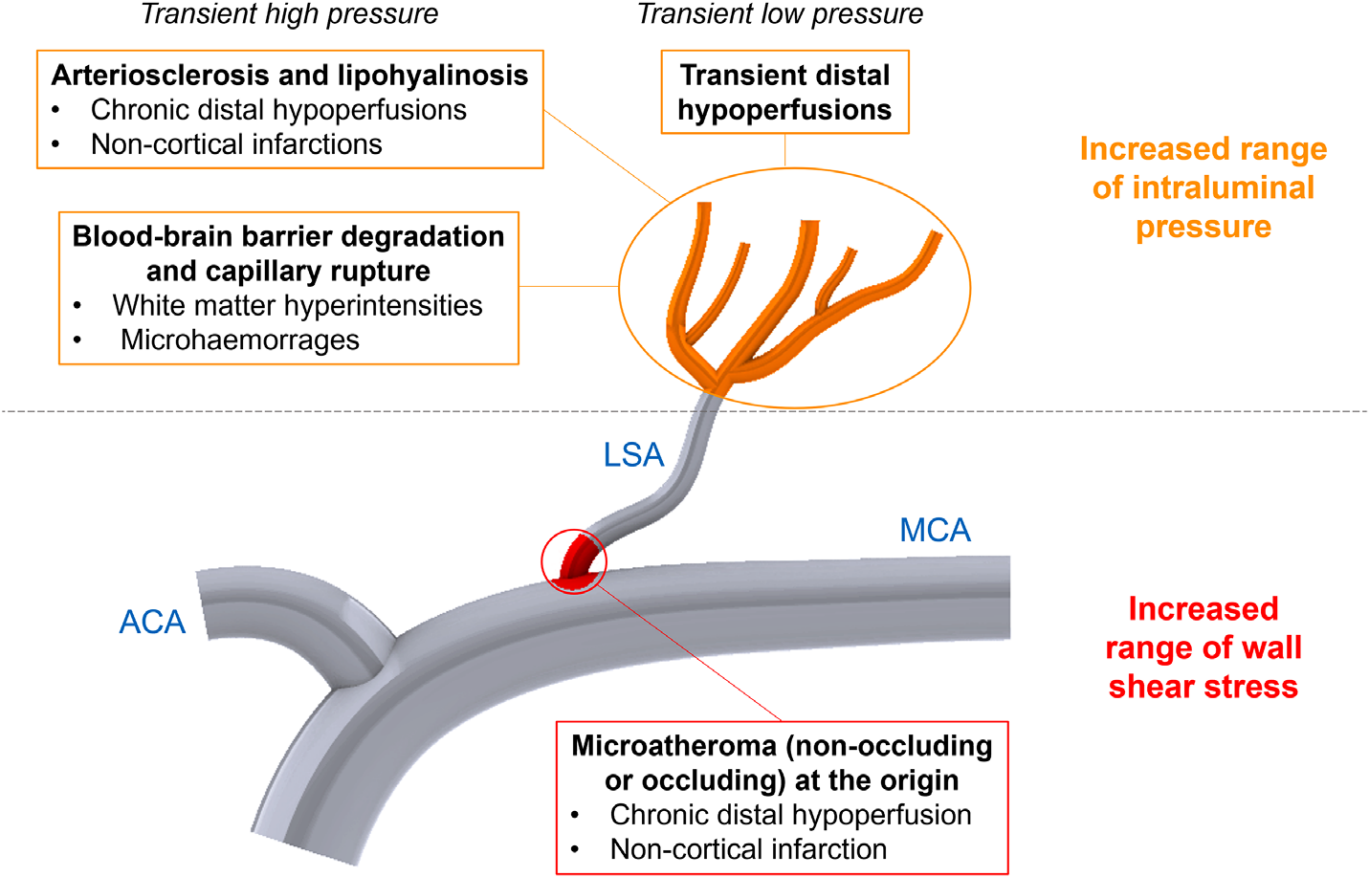
Plausible pathophysiological framework of AF‐related (non‐cardioembolic) hemodynamic effects at the lenticulostriate arteries level.

In conclusion, the present study suggests, for the first time, that the AF exerts a direct detrimental hemodynamic effect on LSAs (Figure 4). This finding supports the hypothesis that, in addition to known risk factors (e.g. hypertension and diabetes), AF may induce per se local hemodynamic perturbations that might promote cerebral SVD and consequently cognitive decline. Further studies are needed to investigate if therapeutic strategies aimed at SR maintenance (antiarrhythmic drugs and/or transcatheter ablation) may reduce cognitive burden by eliminating AF‐induced hemodynamic alterations.

**FIGURE 4:**
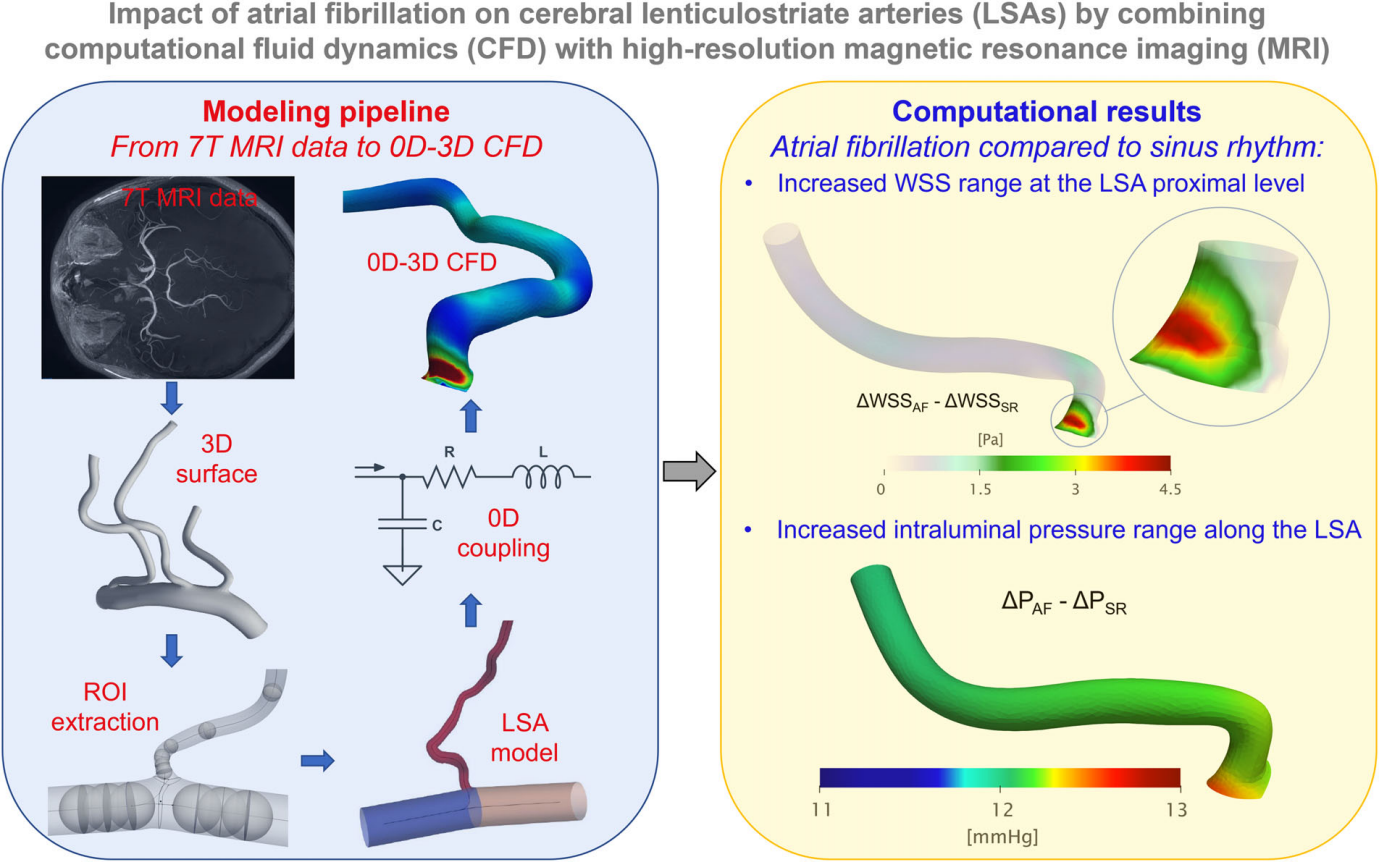
Graphical summary of methodological workflow and results of the study.

## DISCLOSURES

M.A. is a consultant for Biosense Webster and Boston Scientific, a clinical proctor for Medtronic and has received educational fees from Abbott.

## Supporting information

Supporting Information

## Data Availability

Data available on request.
