# Supplementary material for: Atrial fibrillation hemodynamic effects on lenticulostriate arteries identified at 7‐Tesla cerebral magnetic resonance imaging
Source: Clin Transl Med. 2023 Sep 21;13(9):e1367. doi: 10.1002/ctm2.1367 (PMC10514374; doi:10.1002/ctm2.1367)
Supplement: Supplementary file 1 — Supporting Information [file CTM2-13-e1367-s001.docx]

**Atrial fibrillation hemodynamic effects on lenticulostriate arteries identified at 7-Tesla cerebral magnetic resonance imaging**

*Supporting Information*

**Expanded Methods**

## MRI data and geometry reconstruction

Cerebral high-resolution 7T MRI data of 10 subjects aged 19 to 75 years (mean age 45±23 years, 50% males) were obtained from a previously published study from the University Medical Center Utrecht^1^. A 3D T1-weighted magnetization prepared rapid gradient echo sequence, with an acquired resolution of (0.5 x 0.5 x 0.5 mm^3^) and reconstructed resolution of (0.29 x 0.29 x 0.25 mm^3^) was used for the reconstruction of the vasculature geometry.

Geometry reconstruction was carried out following the SimVascular MRI-based modeling pipeline proposed in a previous publication of our group, where a CFD approach was run on the vascular morphology of a single subject^2^. Differently to the present work, where only LSA models extracted from 10 subjects were investigated, in [2] we focused on one (# 2016) of the 10 subjects and analyzed the hemodynamic response of a more extended 3D vascular region, comprising the right internal carotid artery (ICA), the precommunicating ACA, the MCA, and 5 LSAs perpendicularly departing from MCA (four vessels) and ACA (one vessel).

The computational pipeline includes the identification of each vessel pathline, the 2D segmentation of each vessel lumen, the generation of 3D surfaces fitted to groups of 2D segmentations, and the merging of individual vessels to obtain a complete 3D solid model of the vascular district^3,4^. For segmentation purposes, any visible LSA departing from MCA or ACA on both left and right sides was considered.

Each CFD model was composed of one inlet (MCA or ACA) and two outlets (LSA and MCA or ACA). Flow extensions (i.e., cylindrical segments of the same diameter as the section of the vessel where they are applied) were added to the inlet and the outlets to drive away from the domain of interest the surfaces on which boundary conditions are imposed and to uniform the length of individual LSAs. Hence, the distance between the outlet section where pressure condition is set and the LSA-MCA (or LSA-ACA) bifurcation was comparable between the different cases.

## CFD setting: mesh and boundary conditions

In order to perform 3D CFD simulations, blood was assumed as incompressible, homogeneous and Newtonian, with a constant density *ρ*=1060 Kg/m^3^ and a dynamic viscosity *µ*=0.004 Kg/(m · s). All vessels were supposed to be rigid and no-slip condition was set. Under these hypotheses, the blood flow is governed by continuity and Navier-Stokes equations, which were discretized and numerically solved by exploiting the SimVascular simulations tool^2^.

The time step size has been set constant equal to 0.05 ms, while the global maximum edge size (GMES) of the unstructured tetrahedral mesh was chosen after a sensitivity analysis on one of the 17 models, selected as representative. Details of the mesh sensitivity analysis are reported in a following section of this Supporting Information.

The inlet conditions were obtained from the MCA flow rate time series, $Q_{MCA}(t)$, resulting from a combined and validated 0D cardiovascular-cerebral model^5^. The cardiovascular and cerebral models have been previously calibrated and validated in physiological and fibrillated conditions from the central hemodynamics up to the middle cerebral circulation, since definitive clinical data in the cerebral microvasculature are still missing^6–11^. Being able to mimic the heterogeneous cerebrovascular hemodynamics of different pathological conditions, the 0D computational approach was exploited to investigate the effects of cardiac arrhythmias^5,10,12^ and heart rate variability^11^ on the cerebral microcirculation.

Here, the 0D cardiovascular-cerebral model simulates the cerebral hemodynamics in AF and SR conditions at a simulated heart rate of 70 bpm. RR-intervals were artificially built to avoid the patient-specific details (e.g., sex, age, weight, and cardiovascular diseases) inherited by real RR beating, following the algorithm previously proposed in other works^5,7,10^. To focus on AF impact, we considered each of the 17 models, first forced through SR and then by AF rhythm. In brief, being SR a remarkable example of pink noise^13^, RR beats during SR have been extracted from a pink-correlated Gaussian distribution, with mean value µ=0.86 s^7,14^. Standard deviation, σ, is determined considering that the coefficient of variation, cv, lies in the interval [0.05, 0.14] for SR conditions^15^. Thus, cv was kept equal to 0.07 and σ=0.06 s.

The AF beating is commonly unimodal and fully described by the superposition of two statistically independent times, RR = φ + η. φ is taken from a Gaussian distribution and the extraction is based on the correlated pink noise. η is instead drawn from an exponential distribution (with rate parameter γ) and the beating extraction relies on the uncorrelated white noise. The resulting AF beatings are thus represented by an exponentially modified Gaussian distribution with mean value again equal to µ=0.86 s, as in SR condition. The standard deviation value, σ, is determined keeping the coefficient of variation, cv, equal to 0.24^16^. The rate parameter, γ, of the exponential distribution is a linear function of the mean RR (γ = −9.2·RR + 14.6), as proposed in a previous work^17^.

Since these RR intervals are based on beating features^7^ and have been validated and tested over clinically measured beating^13,14,18^, we adopted them as the most suitable and reliable RR time-series to mimic SR and AF beating.

Initially, 5000 beats were simulated through the 0D lumped parameter model in SR and AF. Afterwards, for each beat both maximum, $Q_{max}$, and minimum $Q_{min}$ values were collected over the MCA flow rate time series. In this way, two statistically significant sets of 5000 maximum and minimum values ($Q_{max}$ and $Q_{min}$, respectively) were sampled. Subsequently, the probability density functions, ${p(Q}_{max})$ and ${p(Q}_{min})$, were computed, and the 5^th^ percentile of ${p(Q}_{min})$ and the 95^th^ percentile of ${p(Q}_{max})$ were extracted and forced in all models. In addition, a Poiseuille-like parabolic velocity profile was applied to the entrance. This workflow was performed both in AF and SR conditions at the simulated heart rate of 70 bpm. Therefore, four simulations for each model were run: 5^th^ percentile of ${p(Q}_{min})$ and 95^th^ percentile of ${p(Q}_{max})$, for both AF and SR conditions. Outflow conditions were defined by setting the same pressure $P= P_{0}$ on both outlets downstream the bifurcation, where a workflow analogous to the one used to determine the inlet flow rate was used to obtain $P_{0}$. In fact, starting from the MCA pressure time series $P_{MCA}(t)$ given by the 0D model, four values of $P_{0}$ were extracted: 5^th^ percentile of ${p(P}_{min})$ and 95^th^ percentile of ${p(P}_{max})$, for both AF and SR conditions. Specifically for AF, this allowed us to reproduce the complex flow/pressure oscillation observed during arrhythmia, previously demonstrated to relate to hypoperfusions and hypertensive events in the distal cerebral circle^10^.

## Region of interest extraction

To evaluate the main hemodynamic metrics, several regions of interest (ROIs) from the LSA segments were extracted using the *Vascular Modeling Toolkit* (VMTK). The first step consisted in computing the centerlines of the vascular geometry: weighted shortest paths traced between two extremal points. In order to ensure that the final lines were central to the vessel they belong to, the paths are bound to run on the Voronoi diagram of the vessel model^19^. Secondly, branch splitting was performed. VMTK allows the division of the surface automatically based on geometric considerations, fundamental feature to estimate consistently different morphologies^20^. Finally, branch sections located at a fixed number (*n*) of maximum inscribed spheres from LSAs origin were computed perpendicularly to the branch centerline.

## Hemodynamic parameters investigated

The models provide data on the main hemodynamic variables, such as wall shear stress (*WSS*) and pressure (*P*). For the purposes of this study the focus was set on two hemodynamic parameters: $\Delta WSS$ and $\Delta P$. The first metric was defined as the difference between the WSS magnitude obtained in correspondence of the 95^th^ percentile of ${p(Q}_{max})$ and that at the 5^th^ percentile of ${p(Q}_{min})$:

$$\Delta WSS=\left| \vec{{WSS}_{max}} \right|-\left| \vec{{WSS}_{min}} \right|$$

The same approach was adopted for the pressure, hence:

$$\Delta P=P_{max}-P_{min}$$

These metrics were computed for SR and AF conditions, obtaining two $\Delta WSS$ and $\Delta P$ maps for each model. In order to study the $\Delta WSS$ a ROI with *n*=2 (*n* is the number of inscribed spheres) was computed, while for $\Delta P$ a ROI with *n*=10 was considered. The different choice of *n* for *WSS* and *P* relates to the fact that for WSS the main focus was kept on the proximal portion of the LSA, the most prone area to critical events, while for *P* a greater extension of the LSA vascular wall was investigated. A sensitivity analysis on the extension of the ROIs for both $\Delta WSS$ and $\Delta P$ is reported in a following section of this Supporting Information.

## Mesh sensitivity analysis

Mesh sensitivity analysis was performed by forcing to the inlet a constant flow rate of 3.74 ml/s, which corresponds to the MCA flow rate average both in SR and AF conditions. *WSS* and *P’* (i.e., the difference between the pressure at the i-th point and the average pressure on the LSA outlet section) were evaluated for six different meshes: three uniform meshes with no boundary layers and global max edge size (GMES) of 0.017, 0.0135 and 0.0107 cm and three uniform meshes with the same GMES but three boundary layers (in the boundary layer the density of the mesh in proximity of the vascular wall, where higher velocity gradients are expected, was increased). The time step was set equal to 0.05 ms for all simulations.

The domain considered for the mesh sensitivity analysis was obtained by removing all the existing flow extension (Fig. SM1). As shown in Fig. SM2, mean and standard deviation values were computed, while the percentage relative errors referred to the finest mesh with three boundary layers for all metrics are reported. Given the relative error values, the chosen mesh (GMES = 0.017 cm with 3 boundary layers) turns out to be a good compromise between numerical accuracy and affordable computational cost. As the finest mesh with three boundary layers didn’t lead to significant improvements, the mesh with GMES=0.17 mm with three boundary layers was chosen for the numerical simulations, linearly scaling the GMES according to the radius of the lenticulostriate artery.

## ROI sensitivity analysis

A sensitivity analysis on the extension of the ROIs was performed for both $\Delta WSS$ and $\Delta P$. In particular, *n*=2 and *n*=3 were evaluated for $\Delta WSS$, while *n*=10 and *n*=20 were considered for $\Delta P$. The four different ROIs are shown in figure SM3 for a single representative model.

Tables SM1 and SM2 report, for both AF and SR, the mean and standard deviation values of $\Delta WSS$ and $\Delta P$ averaged on all the 17 models, respectively ($\Delta WSS$: *n*=2 and *n*=3, $\Delta P$: *n*=10 and *n*=20). In addition, Tables SM1 and SM2 show the percentage difference (averaged over all the 17 models) between SR and AF for each metric described above. Since the percentage difference for all the different domains is always considerable and comparable, the ROIs with *n*=2 and *n*=10 were chosen as representative.

## Statistical analysis

The hemodynamic metrics of interest ($\Delta WSS$ and $\Delta P$) within the different ROIs were compared between AF and SR conditions by the Wilcoxon signed rank paired test. More specifically, we checked whether ∆WSS and ∆P mean values for the 17 anatomical models (as shown in Figs. 1B and 2B, and in Tables SM1 and SM2) were significantly different in AF with respect to SR. This statistical analysis was performed using MATLAB software (R2021b) with a significance level of 5%.

**Limitations**

The present study presents the following limiting aspects. First, rigid wall assumption was considered for all CFD analyses, neglecting deformation and compliant effects of the distal cerebral circulation. Second, in order to force all the models with the same flow rate, MCA flow rate was assumed as inlet condition for all the 17 vascular geometries, including the 3 models departing from ACA. Eventually, in order to emphasize AF impact on the LSAs region, the patient-averaged difference between AF versus SR was focused, while the patient-specific link between vessel morphology and LSAs wall shear stress pattern was deliberately not accounted. Such link will need further research to be assessed.

**References of Supporting Information**

1. Bouvy WH, Biessels GJ, Kuijf HJ, Kappelle LJ, Luijten PR, Zwanenburg JJM. Visualization of perivascular spaces and perforating arteries with 7 T magnetic resonance imaging. *Invest Radiol* Invest Radiol; 2014;**49**:307–13.

2. Scarsoglio S, Saglietto A, Tripoli F, Zwanenburg JJM, Biessels GJ, Ferrari GM De, *et al.* Cerebral hemodynamics during atrial fibrillation: Computational fluid dynamics analysis of lenticulostriate arteries using 7 T high-resolution magnetic resonance imaging. *Phys Fluids (1994)* Phys Fluids (1994); 2022;**34**.

3. Updegrove A, Wilson NM, Merkow J, Lan H, Marsden AL, Shadden SC. SimVascular: An Open Source Pipeline for Cardiovascular Simulation. *Ann Biomed Eng* Ann Biomed Eng; 2017;**45**:525–41.

4. Lan H, Updegrove A, Wilson NM, Maher GD, Shadden SC, Marsden AL. A Re-Engineered Software Interface and Workflow for the Open-Source SimVascular Cardiovascular Modeling Package. *J Biomech Eng* J Biomech Eng; 2018;**140**.

5. Saglietto A, Scarsoglio S, Ridolfi L, Gaita F, Anselmino M. Higher ventricular rate during atrial fibrillation relates to increased cerebral hypoperfusions and hypertensive events. *Sci Rep* Nature Publishing Group; 2019;**9**.

6. Korakianitis T, Shi Y. Numerical simulation of cardiovascular dynamics with healthy and diseased heart valves. *J Biomech* J Biomech; 2006;**39**:1964–82.

7. Scarsoglio S, Guala A, Camporeale C, Ridolfi L. Impact of atrial fibrillation on the cardiovascular system through a lumped-parameter approach. *Med Biol Eng Comput* Springer Verlag; 2014;**52**:905–20.

8. Ursino M, Giannessi M. A model of cerebrovascular reactivity including the circle of willis and cortical anastomoses. *Ann Biomed Eng* Ann Biomed Eng; 2010;**38**:955–74.

9. Scarsoglio S, Saglietto A, Gaita F, Ridolfi L, Anselmino M. Computational fluid dynamics modelling of left valvular heart diseases during atrial fibrillation. *PeerJ* PeerJ; 2016;**4**.

10. Anselmino M, Scarsoglio S, Saglietto A, Gaita F, Ridolfi L. Transient cerebral hypoperfusion and hypertensive events during atrial fibrillation: A plausible mechanism for cognitive impairment. *Sci Rep* Nature Publishing Group; 2016;**6**.

11. Scarsoglio S, Ridolfi L. Different Impact of Heart Rate Variability in the Deep Cerebral and Central Hemodynamics at Rest: An in silico Investigation. *Front Neurosci* Front Neurosci; 2021;**15**.

12. Scarsoglio S, Saglietto A, Anselmino M, Gaita F, Ridolfi L. Alteration of cerebrovascular haemodynamic patterns due to atrial fibrillation: An in silico investigation. *J R Soc Interface* Royal Society; 2017;**14**.

13. Hayano J, Yamasaki F, Sakata S, Okada A, Mukai S, Fujinami T. Spectral characteristics of ventricular response to atrial fibrillation. *Am J Physiol* Am J Physiol; 1997;**273**.

14. Hennig T, Maass P, Hayano J, Heinrichs S. Exponential distribution of long heart beat intervals during atrial fibrillation and their relevance for white noise behaviour in power spectrum. *J Biol Phys* J Biol Phys; 2006;**32**:383–92.

15. Goldberger AL, Amaral LA, Glass L, Hausdorff JM, Ivanov PC, Mark RG, *et al.* PhysioBank, PhysioToolkit, and PhysioNet: components of a new research resource for complex physiologic signals. *Circulation* Circulation; 2000;**101**.

16. Tateno K, Glass L. Automatic detection of atrial fibrillation using the coefficient of variation and density histograms of RR and deltaRR intervals. *Med Biol Eng Comput* Med Biol Eng Comput; 2001;**39**:664–71.

17. Anselmino M, Scarsoglio S, Saglietto A, Gaita F, Ridolfi L. A computational study on the relation between resting heart rate and atrial fibrillation hemodynamics under exercise. *PLoS One* Public Library of Science; 2017;**12**.

18. Sosnowski M, Korzeniowska B, Macfarlane PW, Tendera M. Relationship between R-R interval variation and left ventricular function in sinus rhythm and atrial fibrillation as estimated by means of heart rate variability fraction. *Cardiol J* Cardiol J; 2011;**18**:538–45.

19. Piccinelli M, Veneziani A, Steinman DA, Remuzzi A, Antiga L. A framework for geometric analysis of vascular structures: application to cerebral aneurysms. *IEEE Trans Med Imaging* IEEE Trans Med Imaging; 2009;**28**:1141–55.

20. Antiga L, Steinman DA. Robust and objective decomposition and mapping of bifurcating vessels. *IEEE Trans Med Imaging* IEEE Trans Med Imaging; 2004;**23**:704–13.

**Supplementary Figures**

**Figure SM1:** Domain considered for the mesh sensitivity analysis (patient V2016).


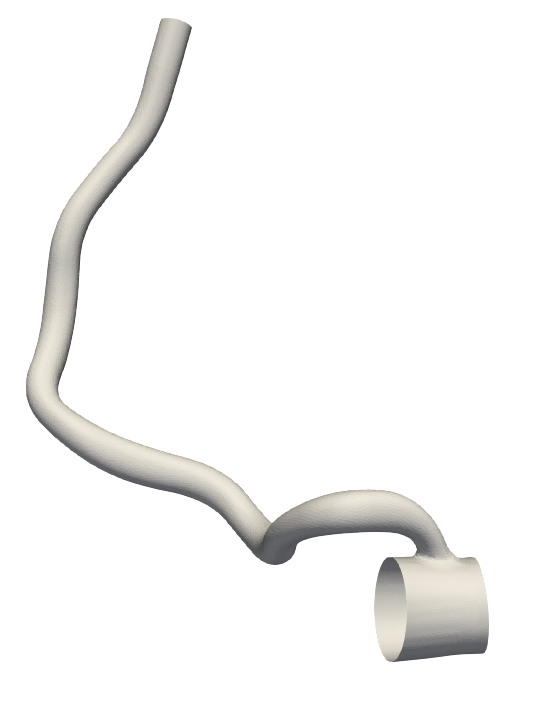


**Figure SM2:** Mesh sensitivity analysis. Top panels: $\mathrm{WSS}$. Bottom panels: $P'$. Mean (left panels) and standard deviation (right panels) values. Six meshes are considered: three uniform meshes with no boundary layers and global max edge size (GMES) of 0.017, 0.0135 and 0.0107 cm and three uniform meshes with the same GMES but three boundary layers. Overbar values are the percentage relative error values referred to the finest mesh with three boundary layers (BL 0.0107).

**
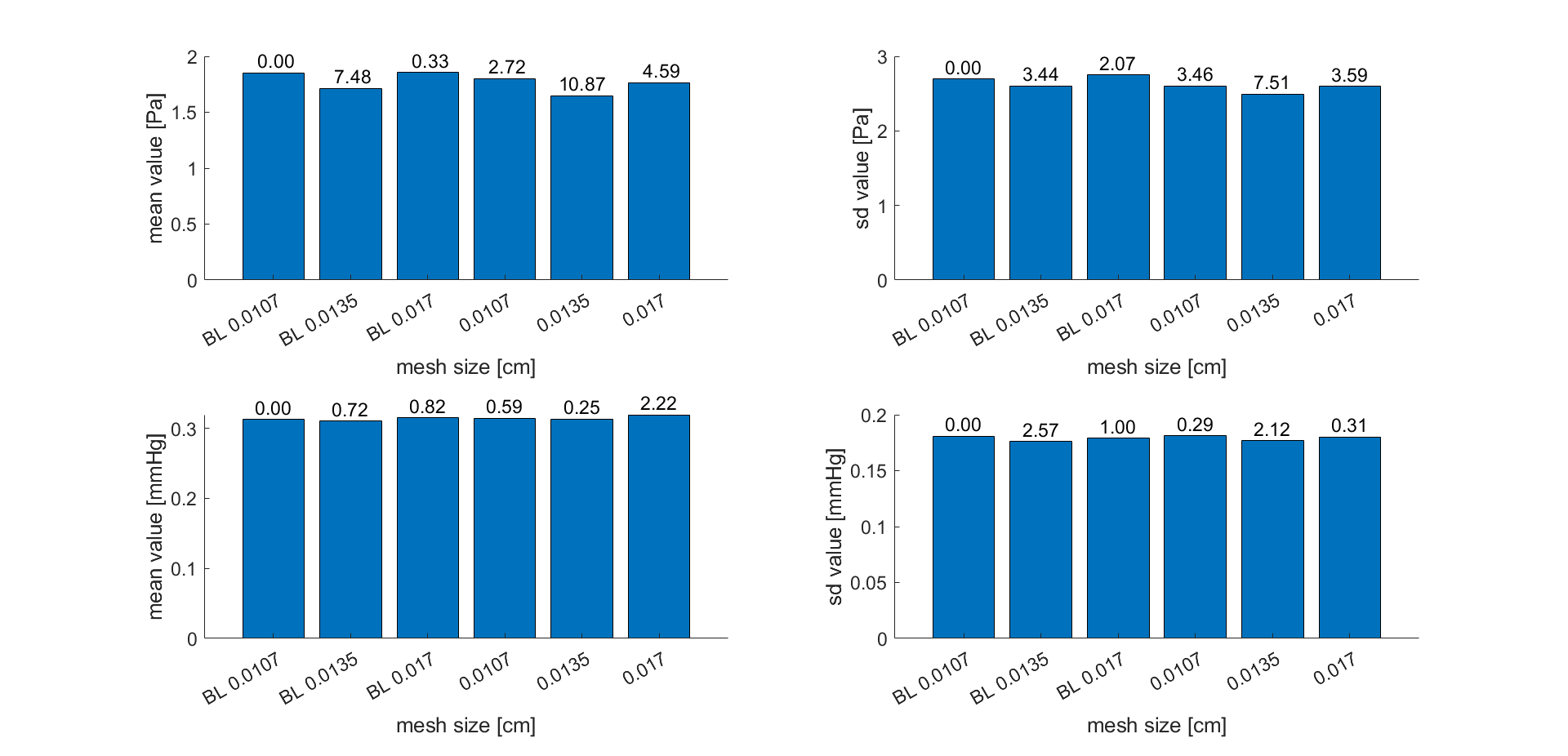
**

**Figure SM3:** CFD models of a representative LSA (patient V2016). a) ROI with *n*=2; b) ROI with *n*=3; c) ROI with *n*=10; d) ROI with *n*=20. In particular, *n*=2 and *n*=3 were evaluated for $\Delta WSS$, while *n*=10 and *n*=20 were considered for $\Delta P$.


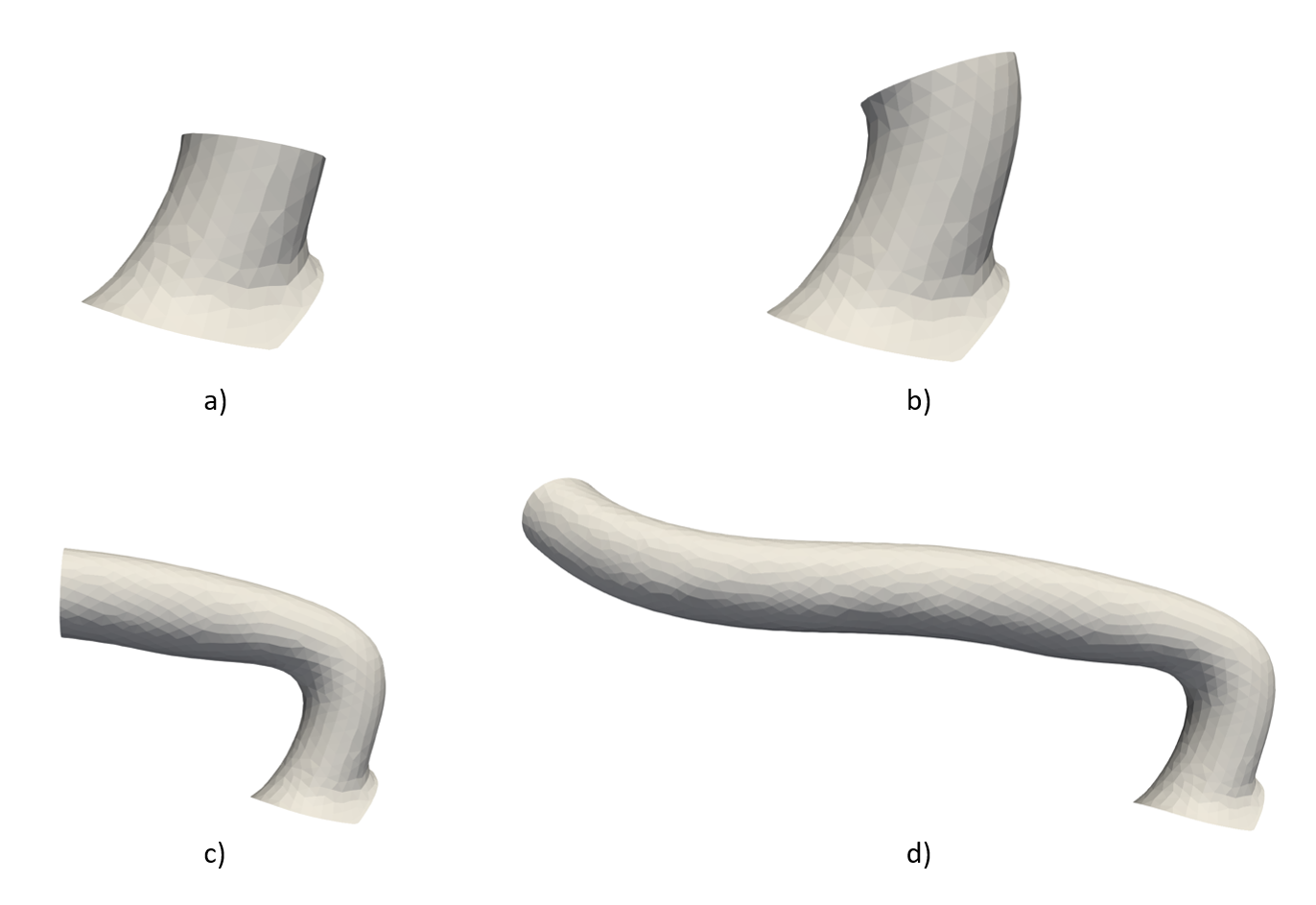


**Figure SM4:** Δ𝑊𝑆𝑆 in SR (left subpanels) and AF (right subpanels) for all the 17 LSA models analyzed, ROI with *n*=2. Δ𝑊𝑆𝑆 values and colorbars are expressed in [Pa].


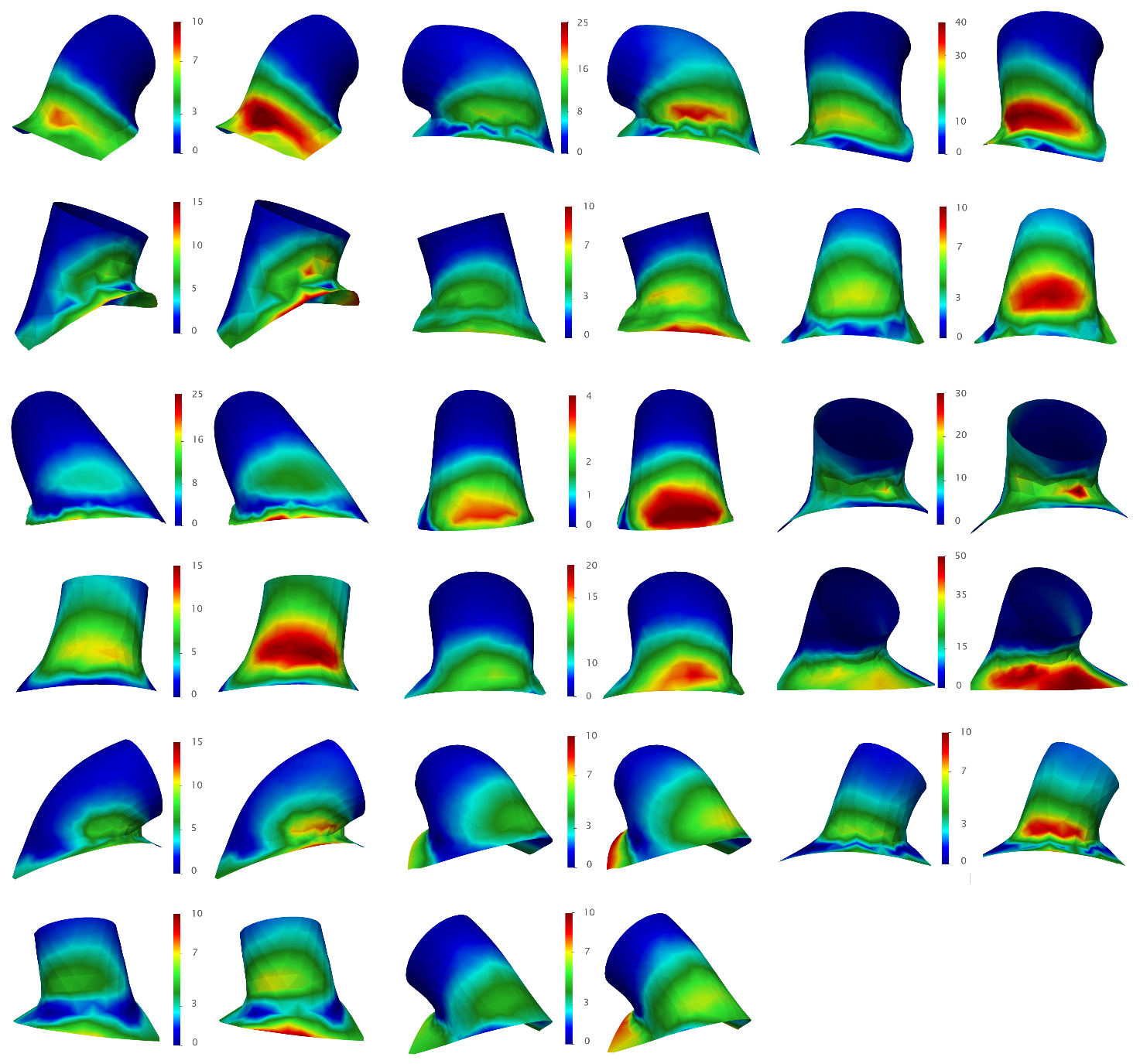


**Supplementary Tables**

**Table SM1:** Mean and standard deviation values of ∆WSS averaged on all the 17 models for each ROI (*n*=2 and *n*=3), in SR and AF conditions.

|  | ROI with n=2 | | ROI with n=3 | |
| --- | --- | --- | --- | --- |
| Heart rhythm | Averaged ∆WSS mean [Pa] | Averaged ∆WSS standard deviation [Pa] | Averaged ∆WSS mean [Pa] | Averaged ∆WSS standard deviation [Pa] |
| SR | 3.07 | 2.87 | 2.68 | 2.68 |
| AF | 4.26 | 3.94 | 3.72 | 3.68 |
| Averaged percentage difference | 37.85 | 36.32 | 37.72 | 36.49 |

**Table SM2:** Mean and standard deviation values of ∆P averaged on all the 17 models for each ROI (*n*=10 and *n*=20), in SR and AF conditions.

|  | ROI with n=10 | | ROI with n=20 | |
| --- | --- | --- | --- | --- |
| Heart rhythm | Averaged ∆P mean [mmHg] | Averaged ∆P standard deviation [mmHg] | Averaged ∆P mean [mmHg] | Averaged ∆P standard deviation [mmHg] |
| SR | 50.37 | 0.11 | 50.28 | 0.14 |
| AF | 62.55 | 0.16 | 62.42 | 0.19 |
| Averaged percentage difference | 24.16 | 38.97 | 24.14 | 36.46 |

**Table SM3. Main clinical characteristics of the included subjects (healthy volunteers).**

| **Subject** | **Age** | **Sex** |
| --- | --- | --- |
| V1988 | 26.1 | Female |
| V2012 | 25.4 | Male |
| V2016 | 19.8 | Male |
| V2019 | 25.9 | Female |
| V2043 | 63.9 | Female |
| V2045 | 27.3 | Male |
| V2066 | 72.7 | Female |
| V2249 | 65.5 | Female |
| V2304 | 51.3 | Male |
| V2363 | 75.5 | Male |
